# The Association of IL-1 and HRAS Gene Polymorphisms with Breast Cancer Susceptibility in a Jordanian Population of Arab Descent: A Genotype–Phenotype Study

**DOI:** 10.3390/cancers12020283

**Published:** 2020-01-23

**Authors:** Laith N. AL-Eitan, Bashar H. Al-Ahmad, Fouad A. Almomani

**Affiliations:** 1 Department of Applied Biological Sciences, Jordan University of Science and Technology, Irbid 22110, Jordan; bmalkawi0@gmail.com (B.H.A.-A.); fouad57@just.edu.jo (F.A.A.); 2 Department of Biotechnology and Genetic Engineering, Jordan University of Science and Technology, Irbid 22110, Jordan

**Keywords:** breast cancer, genetic association, single nucleotide polymorphism, *HRAS*, *IL-1*, cancer susceptibility

## Abstract

Breast cancer (BC) pathogenesis is poorly understood and not yet completely determined. BC susceptibility genes are responsible for 20% to 25% of breast cancer risk. The main objective of this study is to identify the genetic polymorphisms within the Harvey rat sarcoma viral oncogene homolog (HRAS1) and interleukin-1 receptor antagonist (IL1-Ra) genes in Jordanian BC female patients and to investigate the genetic association of these polymorphisms with BC. Samples were collected from 150 Jordanian BC patients and 187 healthy age-matched controls. PCR and PCR-RFLP techniques were used to identify genetic polymorphisms within these candidate genes. The single nucleotide polymorphism single nucleotide polymorphism (SNP) association web tool SNPStats (v. 3.6) was used to investigate the allelic and genotypic association with BC. Different statistical analyses were used to study the correlation between the investigated genetic variants and several prognosis factors of BC. A genetic association between BC susceptibility and *Il-1β* rs1143634 was found specifically at the allelic level of E1 as a risk allele (72% in the cases vs. 64.2% in the controls). Another genetic association was found in the IL-Ra gene (86-VNTR (variable number tandem repeat)), which presented one repeat allele (24.1% in cases vs. 15.59% in controls) and could be considered as a risk allele in Jordanian women. In contrast, this study found that there is no genetic association between Il-1β SNP rs16944 and BC. In addition, a significant association was found between the allelic level of the HRAS1 gene and BC susceptibility. Since this study is the first to be conducted on the genetic susceptibility of these genes to BC in the Jordanian population, more investigations on the link between BC and these variants are recommended to determine the impact of these polymorphisms on other ethnic groups.

## 1. Introduction

Breast cancer (BC) is an accumulation of different malignancies that present in the mammary glands. BC has the highest incidence of all cancers in women worldwide [1]. Among the Jordanian population, BC was ranked number one of the three most common cancers among Jordanian females, as reported by the Jordan Cancer Registry [2]. It was also reported to be the second cause of death after cardiovascular diseases among women [3]. According to the Ministry of Health, BC accounted for nearly 40% of all cancers that affect Jordanian women, with the median age at first diagnosis being 51 [3].

BC etiology is complex as many genes are involved in multiple stages of cancer development [4]. Genetic mutations can lead to BC and have been experimentally linked to some tumor markers, including progesterone receptor (PR), estrogen receptor (ER), and human epidermal growth factor receptor type 2 (HER2) statuses, which are used clinically in the classification of BC [5]. The cellular heterogeneity of BC and many genes involved in regulating cell differentiation, growth, and death highlights the significance of investigating the effects of multiple genetic alterations in BC development and progression [6]. Many candidate gene studies have been performed to identify the genes that contribute to BC development in the Jordanian population. In one study, using mutation analysis, it was found that most screened mutations were present in the *BRCA1* exon 11 gene [7]. Another study found that Jordanians of Circassian descent have a higher risk of BC than their Arab counterparts [8]. Moreover, the 5′UTR polymorphism of the *XRCC* gene was found to be significantly different between Jordanian BC patients and the healthy population [9]. A significant association between *MTHFR* polymorphisms and the incidence of BC in Jordanian women was also found, especially in the 41–60 age demographic [10]. Recently, several genetic association studies of BC have been conducted in Jordanian women [11,12,13,14,15,16,17,18,19] to investigate the genetic association of several candidate genes with BC susceptibility. None of the Jordanian studies have investigated the effect of genetic polymorphisms of the interleukin 1 (*IL-1*) and Harvey rat sarcoma viral oncogene homolog (*HRAS*) genes on the genetic susceptibility of BC. The *IL-1* gene is located on chromosome 2 (q14.1) and encodes the interleukin 1 protein [20,21,22]. This type of protein is considered a cytokine, which, at low concentrations, can affect several types of cells and organs. The IL-1 cytokine has a wide range of roles in adaptive and innate immune responses. Several genetic polymorphisms, such as rs16944 (C/T) and rs1143627 (T/C), within the *IL-1* gene have been reported to play a major role in the genetic susceptibility of deferent cancers [23]. Moreover, the *HRAS* gene is located on chromosome 11 (p15.5) and is considered to be a small monomeric protein with GTPase activity [1,24]. The *HRAS* gene has been the subject of conflicting reports with regard to its role in BC development and progression. It has been reported that increased expression of HRAS is associated with more aggressive breast cancer tumors [25], while in another study, it was observed that HRAS mutations were rarely found in breast cancer tumors [26].

To the best of our knowledge *IL-1* and *HRAS* gene polymorphisms have never been examined to detect their associations with an increased risk of BC among the Jordanian population. Herein, we describe the roles of the variable number tandem repeat (VNTR) polymorphism of the *HRAS* gene and the single nucleotide polymorphism (SNPs) of the *IL-1* gene in Jordanian BC patients.

## 2. Results

### 2.1. Patient Characteristics

Whole pathological reports were obtained for all selected patients. Table 1 shows the major demographic, clinical, and histopathological characteristics of the cohort. A total of 30.8% of the recruited patients had a family history of BC. The minimum age of menopause was 35, and the maximum age was 59, with an average age (±SD) of 47.9 ± 5.3. Also, most of the cases were invasive carcinoma (82.2%), and in 50% of all cases, the lymph nodes were free of tumors. Furthermore, histologic reports for each patient showed that 73% of tumors express the Estrogen Hormone Receptor, 42.8% express the prostergon Hormone Receptor, and only 43.8% express the HER2 receptor.

### 2.2. Hardy–Weinberg Equilibrium (HWE) Test

The Hardy–Weinberg Equilibrium test (HWE) was performed to examine the normal distribution of polymorphisms within *IL-1β* (promoter region and exon5) in both the cases and in the control groups. All the studied SNPs met the HWE standards in both the BC patients and healthy individuals and were included in the current study. The minor alleles of the studied SNPs and their frequencies for both the cases and controls are shown in Table S2.

### 2.3. IL-1 Gene Polymorphisms and their Associations with BC Risk

The frequency distribution of the genotyped polymorphisms (rs16944 within the Il-1β promoter region, rs1143634 within the Il-1β exon 5 region, and 86 bp-VNTR within IL-1Ra) within the IL-1 gene and their associations with BC were evaluated among both BC patients and healthy individuals (Table 2). These genetic associations were conducted using case-control genetic analysis (http://www.quantpsy.org/chisq/chisq.htm) in order to determine if there is a significant difference between the different genotypes and alleles and BC susceptibility, as shown in Table 2.

#### 2.3.1. Genetic Association of rs16944 Polymorphisms with the Risk of BC

**Figure 1:**
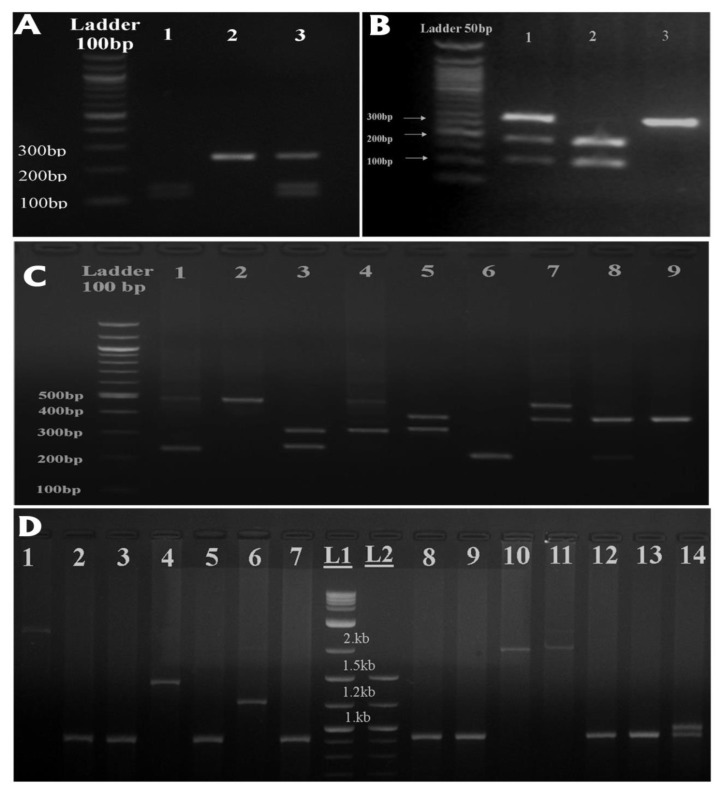
Agarose gel electrophoresis showing different polymorphisms in the *IL-1* and *HRAS* genes: (**A**) PCR-RFLP genotypes in the *IL-1* promoter gene according to the single nucleotide polymorphism (SNP) rs16944. (**B**) PCR-RFLP genotypes in the *IL-1* exon5 gene according to the SNP rs1143634. (**C**) Variable number tandem repeat (VNTR) genotypes in the *IL-1Ra* variable number polymorphism with 86 bp repeats. (**D**) VNTR genotypes in the *HRAS1* gene variable number polymorphism with 28 bp repeats.

The genotypes of the *Il-1β* rs16944 SNP were analyzed using PCR-RFLP and subsequent gel electrophoresis. Figure 1A shows the different genotypes using gel electrophoresis. Three genotypes were detected based on their band sizes. Homozygous (C/C) genotype and homozygous (T/T) genotypes were present at sizes of 190 + 114 bp and 304 bp, respectively, while the presence of heterozygous alleles (C/T) was indicated by the appearance of both bands (190 bp + 114 bp and 304 bp).

The genotype frequencies of these polymorphisms are presented in Table 2. The results of this study show no significant differences between the cases and the healthy control with a chi-square = 1.364 and a *p*-value = 0.521, with an odds ratio and 95% CI, C/C = 1, C/T = 1.02 (0.64–1.64), and TT = 1.42 (0.67–2.63). Moreover, in allelic frequency, the BC patients did not significantly differ from the healthy individuals with a chi-square = 0.986 and a *p*-value = 0.342, with an odds ratio and 95% CI, C = 1.064 (0.941–1.204), T = 0.909 (0.754–1.097). The homozygous TT allele was more frequent in the cases (19.9%) than in the controls (15%), while the CC allele and heterozygous CT allele were less frequent in the cases (37.1% and 43%) than in the healthy controls (39.8% and 45.2%), respectively. At the allelic level, the C allele was less frequent in the cases (58.6%) than in the healthy control (62.4%), while the T allele was more frequent in the cases (41.4%) than in the control (37.6%), which indicates that the T allele and the TT genotype were more prevalent in the BC in the current study.

#### 2.3.2. Genetic Association of rs1143634 with the Risk of BC

The second SNP in the same cluster within the Il-1β gene is rs1143634, which was analyzed using PCR-RFLP. Figure 1B presents the results of different genotypes using gel electrophoresis. In total, three genotypes were detected: a homozygous (E1/E1) genotype at a size of 135 + 114 bp and a homozygous (E2/E2) genotype at 249 bp, while the heterozygous allele (E1/E2) presented both bands (135 + 114 bp, 249 bp).

This study found that the rs1143634 differs between BC patients and healthy individuals, as shown in Table 2. This polymorphism has an odds ratio and 95% CI (E1/E1 = 1, E1/E2 = 0.59 (0.37–0.93), and E2/E2 = 0.59 (0.27–1.26)). Additionally, the allele frequencies between the BC patients and healthy individuals were significant with a chi-square = 4.577 and a *p*-value = 0.038, with an odds ratio and 95% CI (E1 = 0.892 (0.804–0.989), E2 = 1.278 (1.018–1.604)). For example, the heterozygous allele E1/E2 was more frequent in the healthy individuals (49.2%) than in the studied BC patients (38.5%), while the homozygous alleles E1/E1 and E2/E2 were more frequent in the BC patients (52.7% and 8.8%) than in the healthy individuals (39.6% and 11.2%), respectively. At the allelic level, the E1 allele was more frequent in BC patients (72%) than in healthy individuals (64.2%), while the E2 allele was less frequent in BC patients (28%) than in healthy individuals (35.8%). These results indicate that the risk allele for BC patients in this study could be the E1 allele.

#### 2.3.3. Genetic Association of IL-1Ra 86bp-VNTR with the Risk of BC

The genotypes of the 86 bp-VNTR were analyzed using PCR followed by gel electrophoresis. Figure 1C shows the different genotypes using agarose gel electrophoresis. Nine different genotypes were detected according to the number of repeats, composed of the following alleles: Allele (1) was present at a size of 240 bp, allele (2) at 325 bp, allele (3) at 410 bp, and allele (4) at 500 bp. All the homozygous genotypes had the same allele size (1/1, 3/3, 4/4), while the heterozygous genotype had two different alleles (1/2, 1/3, 1/4, 2/3, 2/4, 3/4).

The genotype frequencies of 86 bp-VNTR showed the highest association with BC susceptibility. Further, the genotype frequency in the BC patient group differed significantly from that of the healthy individuals, with a *p*-value = 0.014. In addition, the allele frequencies between the BC patients and healthy individuals were significantly different, with a *p*-value = 0.0003, as shown in Table 2. Most of the repeats were not different between the BC patients (65.1%) and healthy individuals (48.3%), except for the 3/3 repeat. However, the 1/1 repeat at the genotypic level and the 1 repeat at the allelic level are significantly associated with a risk of BC.

### 2.4. Genetic Association of the HRAS1 28bp-VNTR Polymorphism with the Risk of BC

A total of four common alleles (a1, a2, a3, and a4) were identified to correspond to PCR product sizes of 924 bp, 1300 bp, 1900bp, and 2350 bp, respectively, while the rare HRAS1 VNTR alleles were shown to be the increment or decrement of one or more repeat motifs (28 bp) from the four common alleles. Figure 1D shows some common and rare genotypes of the 28bp-VNTR polymorphism.

Table 2 shows the common/common, common/rare, and rare/rare genotypes. These genotype frequencies were not significantly different between the BC patients and healthy individuals (*p*-value = 0.812, OR (95% CI), C/C = 1 (not applicable), C/R = 1.11 (0.58–2.14) and R/R = 0.82 (0.40–1.68)). This study also shows that the CC and CR genotypes were slightly more frequent in BC patients (67.7% and 13.7%) than in healthy individuals (67.1% and 12.2%). In contrast, the RR genotype was less frequent in the cases (9.6%) than in the control (11.7%). The rare and common allele frequencies of the BC patients and healthy individuals are shown in Table 3. These results show a significant association with BC at the allelic level, with an estimated overall *p*-value = 0.022. The current study shows that the most frequent allele within common alleles is A1 in both BC patients (81.5%) and healthy individuals (78.6%), while the most frequent rare allele is A1+2 within BC patients (4.5%) and healthy individuals (4.2%).

### 2.5. Association between the IL-1 Cluster and HRAS1 Gene Polymorphisms and the Clinico–Pathological Characteristics of Breast Cancer (BC)

We investigated the association between the polymorphisms of the IL-1 and HRAS1 genes and clinical and pathological characteristics of BC. The results are shown in Table 4 with the estimated *p*-value for each clinical characteristic. We detected five significant *p*-values that correlated these polymorphisms with age at menopause, co-morbidity, tumor differentiation, tumor stage, and breastfeeding status (0.02 at IL-1Ra, <0.0001 at HRAS1, <0.0001 at rs1143634, 0.016 at IL-1Ra, and 0.044 at HRAS1, respectively).

## 3. Discussion

The candidate IL-1 (rs16944, rs1143634, 86bp-VNTR) and HRAS1 (28bp-VNTR) gene polymorphisms have been reported to play a major role in BC risk. These polymorphisms were chosen according to their significant functional relevance and their location inside the gene [27,28].

This study was designed to examine the genetic association between the candidate gene polymorphisms and BC susceptibility in the Jordanian population. This study is considered the first study of its kind to describe and detect the association between *IL-1* and *HRAS1* SNP and VNTR polymorphisms and BC in a Jordanian population of Arab descent. Our results illustrate that there is a highly significant association between both the *IL-1Ra* gene within the second intron region and the *IL-1* exon5 +3953 and the risk of BC among Jordanian Arabs.

The genes encoding the *IL-1* family are mapped on chromosome 2q14 and are composed of two cytokines, *IL-1* alpha (*IL-α*) and IL-1 beta (*IL-β*), in addition to the *IL-1Ra* receptor antagonist. Our target in this gene was the following cluster: *Il-1β* promoter, exon5, and *IL-1Ra*. In the Caucasian population, no significant association was found between both the *IL-1RN* and *IL-1beta* genes and the risk of BC within them, except for the 511 C/T polymorphisms in the interleukin 1 beta (IL1B) promoter region polymorphism, which was related to the tumor stage and lymph node involvement phenotypes [29]. Moreover, a significant association of the IL-1RN (variable number 240/410 alleles) polymorphism with the risk of malignant BC was reported in Indian women [22]. In the present study, we described a group of clinical and pathological features that might be associated with increased BC risk among Jordanian women. Five significant *p*-values were detected for the age at menopause, co-morbidity, tumor differentiation, tumor stage, and breastfeeding status, which had *p*-values of 0.02 at *IL-1Ra*, <0.0001 at *HRAS1*, <0.0001 at *rs1143634*, 0.016 at *IL-1Ra*, and 0.044 at *HRAS1*.

A genetic association was found between BC susceptibility and the rs1143634 SNP, which is located in exon 5 of the Il-1β gene, specifically at the allelic level of E1 as a risk allele (72% in cases vs. 64.2% in controls). Another significant association was found within this cluster for the 86bp-VNTR of the IL Ra gene with repeat number 1 (24.1% in the cases vs. 15.59% in the controls) as a risk allele, which has the highest association with BC risk among the studied polymorphisms. These results confirm the strong role of these polymorphisms in the risk of BC—especially the 1/1 repeat genotype and 1 repeat allele. The frequency and distribution of the genetic polymorphisms of the IL-1 gene cluster (Il-1β promoter, exon5, and IL-1Ra) within other populations in comparison to the Jordanian population are presented in Tables S3–S5. In terms of the genotypic frequencies of the Il-1β gene polymorphisms (as illustrated in Table S3 and in accordance with the other population data), there is no association between the Il-1β promoter polymorphism and the risk of BC. These findings are very similar to those of the previous works that have been conducted on Caucasian populations in Germany and in the UK.

Moreover, the current study found that the results of this research in regards to the Il-1β exon5 polymorphism are similar to those of the Germany Caucasian study (Table S4), with a *p*-value 0.057 at the genotypic level. In addition, the results in our study were significantly associated with the risk of BC (*p*-value = 0.0003) when we compared the (E1) and (E2) allele frequencies of the IL-1Ra gene polymorphism between BC patients and healthy individuals.

The present study reported that the IL-1Ra gene polymorphism has the strongest statistical association with BC, which is similar to the results obtained by the Korean study but was not similar to the Caucasian and Indian studies (Table S5).

Finally, no genetic association was found between the HRAS rare genotypes and BC risk in the current Jordanian study. However at the allelic level, there is a significant association between the a3 and a4 alleles and the risk of BC [24]. Correspondingly, it was determined that rare alleles in the *HRAS* VNTR were significantly associated with the risk of BC [1].

In agreement with previous data and studies, our results showed that there was no association between HRAS1 and BC at the genotypic level, but, in contrast, HRAS1 was significantly associated with BC susceptibility at the allelic level. There was a significant association with BC susceptibility at the allelic level, especially for the A1 allele, with an overall *p*-value = 0.022. Moreover, allele A1 is considered to be a risk allele associated with BC. The frequency of the HRAS1 28-VNTR variant compared to other populations is illustrated in Table S6. Following the Caucasian population (among whom there is an association at the allelic level between the risk of BC and genetic polymorphisms within the HRAS1 gene), our study also showed a significant association of HRAS1 gene variants with the risk of BC only at the allelic level.

## 4. Materials and Methods

### 4.1. Studied Population

A total of 150 females recruited from the Breast Surgery Clinic in the Royal Medical Services (RMS) were randomly selected for a retrospective study. In addition, 187 unrelated healthy Jordanian females with no breast cancer history were used as the control. The control group was recruited from the blood bank at the RMS. The average age (±SD) of the cohort was 53.1 ± 13.1 years, with a median of 51 and a range of (22 to 95). The average age (±SD) was 36.6 ± 10.9 years with a median of 37 (ranging from (18 to 73) years) for the control group. This study was established according to the provisions of the Human Ethics Standard of Jordan University of Science and Technology and in compliance with the Intuitional Review Board (IRB) Guidelines at the Jordan University of Science and Technology. Approval for the patient’s recruitment, including blood samples and clinical data collection, were also obtained from the Human Ethics Committee at the (RMS). In addition, written informed consent was obtained from all subjects in the study. Each participant was informed about why this study was being conducted, why clinical data were obtained from the medical records, and what the aims, benefits, and details of this study were.

### 4.2. DNA Extraction and Quantification of Isolated Genomic DNA

Blood samples were previously collected into EDTA tubes (5 mL) at the (RMS) laboratories, from both the patients and their matched healthy controls. Extraction of genomic DNA was performed using a Gentra^®^ Puregene^®^ Blood Kit (Qiagen, Germany) following the manufacturer’s protocol. The DNA concentration (ng/µL) and purity (A260/280) was measured by a Nano-Drop ND-1000 UV-Vis Spectrophotometer (BioDrop, UK), and the blank was 1.5 µL of DNA rehydration solution. The DNA concentration was calculated using the c = (A*e)/b equation, where c is the nucleic acid concentration (ng/µL), A is the absorbance, e is the wavelength-dependent extinction coefficient in ng–cm/µL, and b is the path length in cm.

### 4.3. DNA Genotyping

The polymerase chain reaction (PCR) and restriction fragment length polymorphism (RFLP) techniques were used to identify genetic polymorphisms within these candidate genes. PCR was performed using a Veriti Thermal Cycler (Applied Biosystems, Foster City, CA, USA.)). The program details are shown in Table S1. The separation of PCR products of the IL-1Ra intron 2 gene was performed using a 2% agarose gel. Similarly, PCR products of the region with the VNTR polymorphism in the HRAS1 gene were separated with a 1% agarose gel. Bands were observed using ultraviolet illumination, and images of the gels were captured by an AlphaImager^®^ Mini system (Protein Simple, San Jose, CA, USA) [26,27].

### 4.4. DNA Genotyping Using PCR-RFLP

#### 4.4.1. IL-1β rs16944 SNP Genotyping

The allele of the SNP was determined using the RFLP technique. Briefly, the PCR reaction, PCR program, primers, and annealing temperatures are shown in Table S1. A total of 10 μL of PCR product was added to 10 μL restriction enzyme (0.2 μL Ava1 enzyme), 1μL cut smart buffer, and 8.8 μL nuclease-free water. The mixture was spun down and then incubated for 5 h at 65 °C. Next, 10 μL of each digested PCR product was separated by electrophoresis with a 2% agarose gel. Bands were visualized using ultraviolet illumination, and images of the gels were taken by an AlphaImager^®^ Mini system (Protein Simple, San Jose, CA, USA) [26].

#### 4.4.2. IL-1β (rs1143634) and SNP genotyping

The genotypes of rs1143634 SNP were determined by the PCR technique, as described previously in [26]. The PCR program, primers, and annealing temperatures are summarized in Table S1 [26].

### 4.5. Statistical Analysis

The SNP association web tool snpStats (v. 3.6) was used to count allele frequencies. In addition, SNPStats was also used to calculate the genotype frequency for each allele using the Hardy–Weinberg equilibrium equation (p2 + 2pq + q2 = 1). In order to define the *p*-values for allele and genotypic association, a Chi-square test (Pearson χ2 test) and ANOVA test were used (http://www.quantpsy.org/chisq/chisq.htm) to determine if there are significant differences between the expected, observed, and calculated genotype frequencies between BC patients and healthy individuals. A *p*-value < 0.050 was considered to be statically significant.

### 4.6. Ethics Committee Approval and Patient Consent

Ethical approval was obtained from the Intuitional Review Board (IRB), with ethical code number 32/104/2017 at the Jordan University of Science and Technology. Written informed consent was obtained from all participants. In this study, the collected samples were obtained from the Jordanian Royal Medical Services (JRMS).

## 5. Conclusions

To the best of our Knowledge, this study is the first of its kind to investigate the genetic association of IL-1 (rs16944, rs1143634, IL-Ira 86bp VNTR) and HRAS1 (28bp-VNTR) gene polymorphisms with the risk of BC in a Jordanian population of Arab descent. We found that there is a genetic association between BC susceptibility and he IL-1Ra gene VNTR (24.1% in cases vs. 15.59% in controls), with repeat number 1 as a risk allele. Moreover, this study revealed that there is a significant association at the allelic level of rs1143634 SNP within the *Il-1β* gene, with E1 as a risk allele (72% in cases vs. 64.2% in controls). In contrast, there is no genetic association between rs16944 SNP within the *Il-1β* gene and the risk of BC. In addition, a significant genetic association with the risk of BC was also found for the 28bp-VNTR within the *HRAS1* gene, specifically with the A1 allele.

Finally, it is obvious that BC pathogenesis in Jordanian Arab women is predisposed to different genetic factors compared to that in other ethnicities. While all of the genetic polymorphisms included in this study have been previously suggested to play a major role in BC among other ethnicities, only rs16944 and IL-1Ra 86bp VNTR showed a significant association with BC or its risk and prognosis in Jordanians. As few genetic association and pharmacogenomic studies have been conducted in Jordan [30,31], a multi-center and multi-ethnic approach should be applied in future studies to build a genetic landscape profile for BC among Jordanian women.

## Figures and Tables

**Table 1:** Clinical and pathological characteristics for selected breast cancer patients.

Clinical Characteristics		Frequency N (%)	Pathological Characteristics		Frequency N (%)
Body mass index (BMI)	≤25	36 (24.6%)	Progesterone receptor	Positive	57 (42.8%)
	>25	110 (75.4%)		Negative	76 (57.2%)
First pregnancy (age)	<20	38 (30.4%)	Estrogen receptor	Positive	95 (73%)
	≥20	87 (69.6%)		Negative	35 (27%)
Age at breast cancer diagnosis	<45	50 (34.2%)	Tumor differentiation	Low. differentiation	43 (32.5%)
	≥45	96 (75.8%)		Mid and High. differentiation	89 (67.5%)
Age at first menstruation	<13	45 (31.1%)	Axillary lymph nodes	Free of tumor	72 (50%)
	≥13	100 (68.9%)		Show metastatic Carcinoma	72 (50%)
Breastfeeding status	Yes	91 (62.8%)	Tumor stage	PT1-PT2	127 (93.3%)
	No	54 (37.2%)		PT3-PT4	9 (6.7%)
Age at menopause	<50	37 (56.1%)	Histology classification	in situ carcinoma	24 (17.7%)
	≥50	29 (43.9%)		invasive carcinoma	111 (82.2%)
Family history	Yes	45 (31%)	Tumor size	≤2 cm	36 (26.9%)
	No	101 (69%)		2 < x ≤ 5	53 (39.5%)
Allergy	Yes	37 (25.3%)		>5	45 (33.6%)
	No	109 (74.7%)	Lymph node involvement	Yes	121 (84%)
Smoking	Yes	38 (26.9%)		No	23 (16%)
	No	103 (73.1%)	*Human* epidermal growth factor receptor 2 (Her2) marker	Positive	43 (43.9%)
Co-morbidity	Yes	68 (47.2%)		Negative	55 (56.1%)
	No	75 (52.8%)			

*p*-value < 0.05 is considered to be significant.

**Table 2:** Distribution and association of the investigated IL-1 gene cluster (Il-1β promoter, exon5, and IL-1Ra) and *HRAS1* gene polymorphisms and breast cancer (BC).

Polymorphism	Allelic and Genotypic Frequencies in the Cases and Controls				
	Allele/Genotype	Cases (*n* = 150)	Controls (*n*= 188)	*p*-value *	Chi-Square
Il-1β promoter rs16944	C	177 (58.6%)	232 (62.4%)	0.342	0.986
	T	125 (41.4%)	140 (37.6%)		
	CC	56 (37.1%)	74 (39.8%)	0.521	1.364
	CT	65 (43.0%)	84 (45.2%)		
	TT	30 (19.9%)	28 (15%)		
Il-1β exon5 rs1143634	E1	213 (72.0%)	240 (64.2%)	0.038	4.577
	E2	83 (28.0%)	134 (35.8%)		
	E1E1	78 (52.7%)	74 (39.6%)	0.057	5.747
	E1E2	57 (38.5%)	92 (49.2%)		
IL-1RA 86bp-VNTR	1	70 (24.1%)	58 (15.59%)	0.0003	18.258
	2	8 (2.7%)	1 (0.2%)		
	3	196 (67.5%)	299 (80.3%)		
	4	16 (5.51%)	14 (3.76%)		
	1/1	9 (6.2%)	5 (2.7%)	0.014	18.364
	3/3	70 (48.3%)	121 (65.1%)		
	4/4	2 (1.4%)	1 (0.5%)		
	1/2	2 (1.4%)	0 (0%)		
	1/3	46 (31.7%)	46 (24.7%)		
	1/4	4 (2.8%)	2 (1.1)		
	2/3	4 (2.8%)	1 (0.5%)		
	2/4	2 (1.4%)	0 (0%)		
	3/4	6 (4.1%)	9 (4.8%)		
HRAS1 gene 28bp-VNTR	CC *	112 (76.7%)	137 (76.1%)	0.812	0.464
	CR *	20 (13.7%)	22 (12.2%)		
	RR *	14 (9.6%)	21 (11.7%)		

*p*-value < 0.05 is considered to be significant, CC *: common/common alleles, CR: common/rare alleles, RR: rare/rare alleles.

**Table 3:** Distribution and association of the investigated *HRAS1* VNTR and breast cancer (BC).

HRAS1 Alleles	Cases	Controls	*p*-value
Common Alleles			
A1	238 (81.5%)	283 (78.6%)	0.368
A2	6 (2.1%)	11 (3.1%)	0.423
A3	2 (0.7%)	0	0.109
A4	1 (0.3%)	2 (0.6%)	0.689
Rare Alleles			
A1+2	13 (4.5%)	15 (4.2%)	0.841
A1+3	2 (0.7%)	3 (0.8%)	0.841
A1+4	5 (1.7%)	0	0.011
A1+5	0	1 (0.3%)	0.368
A1+6	2 (0.7%)	0	0.109
A1−1	4 (1.4%)	5 (1.4%)	1
A1−2	2 (0.7%)	8 (2.2%)	0.109
A1−5	0	1 (0.3%)	0.368
A2+2	0	5 (1.4%)	0.045
A2+3	9 (3.1%)	9 (2.5%)	0.61
A2+4	1 (0.3%)	0	0.31
A2+6	1 (0.3%)	1 (0.3%)	0.920
A3+3	2 (0.7%)	0	0.109
A3+4	2 (0.7%)	4 (1.1%)	0.548
A3+6	0	1 (0.3%)	0.368
A4+3	1 (0.3%)	8 (2.2%)	0.045
A4+4	1 (0.3%)	0	0.31
A4+5	0	2 (0.6%)	0.193
A4+6	0	1 (0.3%)	0.368

**Table 4:** The association between different IL-1 clusters and HRAS1 gene polymorphism genotypes and the clinico–pathological characteristics of breast cancer (BC).

Clinical Characteristics	Il-1gene Cluster			HRAS1gene
	rs:16944	rs1143634	IL-1R 86 bp-VNTR	28bp-VNTR
**Body mass index**	0.802 ^a^	0.885 ^a^	0.944 ^a^	0.341 ^a^
	0.221 ^c^	0.122 ^c^	0350 ^c^	1.084 ^c^
**Age at first pregnancy**	0.414 ^a^	0.188 ^a^	0.945 ^a^	0.498 ^a^
	0.889 ^c^	1.694 ^c^	0.375 ^c^	0.701 ^c^
**Age at BC diagnosis**	0.344 ^a^	0.266 ^a^	0.646 ^a^	0.308 ^a^
	1.08 ^c^	1.337 ^c^	0.769 ^c^	1.186 ^c^
**Allergy**	0.980 ^a^	0.866 ^a^	0.178 ^a^	0.481 ^a^
	0.040 ^b^	0.122 ^c^	11.44 ^b^	1.464 ^b^
**Age at menarche**	0.957 ^a^	0.347 ^a^	0.765 ^a^	0.577 ^a^
	0.044 ^c^	1.068 ^c^	0.614 ^c^	0.553 ^c^
**Breastfeeding status**	0.206 ^c^	0.285 ^a^	0.786 ^a^	0.044 ^a^
	3.159 ^b^	2.508 ^b^	4.726 ^b^	6.264 ^b^
**Age at menopause**	0.364 ^a^	0.908 ^a^	0.02 ^a^	0.659 ^a^
	1.027 ^c^	0.097 ^c^	2.791 ^c^	0.420 ^c^
**Family history**	0.835 ^a^	0.416 ^a^	0.489 ^a^	0.323 ^a^
	0.361 ^b^	1.752 ^b^	7.448 ^b^	2.257 ^b^
**Co-morbidity**	0.452 ^a^	0.834 ^a^	0.438 ^a^	<0.0001 ^a^
	1.588 ^b^	0.363 ^b^	7.957 ^b^	151.2 ^b^
**Smoking**	0.369 ^a^	0.960 ^a^	0.327 ^a^	0.153 ^a^
	0.127 ^b^	0.81 ^b^	9.182 ^b^	3.757 ^b^
**Progesterone receptor status**	0.425 ^a^	0.864 ^a^	0.605 ^a^	0.491 ^a^
	1.713 ^b^	0.293 ^b^	6.377 ^b^	1.424 ^b^
**Estrogen receptor status**	0.940 ^a^	0.179 ^a^	0.184 ^a^	0.433 ^a^
	0.124 ^b^	3.345 ^b^	11.32 ^b^	18.35 ^b^
**HER2**	0.207 ^a^	0.649 ^a^	0.750 ^a^	0.633 ^a^
	3.150 ^b^	0.865 ^b^	5.071 ^b^	0.914 ^b^
**Tumor differentiation**	0.697 ^a^	<0.0001 ^a^	0.160 ^a^	0.304 ^a^
	0.722 ^b^	138.5 ^b^	10.53 ^b^	10.59 ^b^
**Axillary lymph nodes**	0.505 ^a^	0.677 ^a^	0.316 ^a^	0.930 ^a^
	1.367 ^b^	0.779 ^b^	9.322 ^b^	0.146 ^b^
**Tumor stage**	0.797 ^a^	0.459 ^a^	0.016 ^a^	0.606 ^a^
	0.454 ^b^	1.559 ^b^	18.80 ^b^	1.627 ^b^
**Histology classification**	0.949 ^a^	0.721 ^a^	0.281 ^a^	0.347 ^a^
	0.104 ^b^	0.655 ^b^	9.781 ^b^	2.119 ^b^
**Tumor size**	0.529 ^a^	0.924 ^a^	0.901 ^a^	0.519 ^a^
	0.640 ^c^	0.079 ^c^	0.429 ^c^	0.659 ^c^
**Lymph node involvement**	0.794 ^a^	0.807 ^a^	0.180 ^a^	0.989 ^a^
	0.460 ^b^	1.612 ^b^	11.39 ^b^	0.311 ^b^

^a^ A *p*-Value < 0.05 is considered to be significant; ^b^ A Pearson’s chi-squared test was used to determine the genotype–phenotype association; ^c^ An Analysis of variance (ANOVA) test was used to determine the genotype–phenotype association.

## Supplementary Materials

The following are available online at https://www.mdpi.com/2072-6694/12/2/283/s1, Table S1: Primers, annealing temperatures, PCR product sizes and restriction enzymes that were used for genotyping of *IL-1* (rs16944, rs1143634, IL-Ira 86bp VNTR) and *HRAS1* (28bp-VNTR) gene. Table S2: Il-1β gene polymorphisms and their minor allele frequencies, and HWE p-values in cases and control. Table S3: Frequency of IL1B promoter gene polymorphisms in different populations. Table S4: Frequency of IL1B exon 5 gene polymorphisms in different populations. Table S5: Genetic association studies of IL-1Ra gene polymorphisms in different populations. Table S6: Frequency of HRAS1 gene polymorphisms in different populations.
