# Supplementary material for: The Association of IL-1 and HRAS Gene Polymorphisms with Breast Cancer Susceptibility in a Jordanian Population of Arab Descent: A Genotype–Phenotype Study
Source: Cancers (Basel). 2020 Jan 23;12(2):283. doi: 10.3390/cancers12020283 (PMC7073163; doi:10.3390/cancers12020283)
Supplement: Supplementary file 1 [file cancers-12-00283-s001.pdf]

Supplementary material

Table S1: Primers, annealing temperatures, PCR product sizes and restriction enzymes that were used for genotyping of *IL-1* (rs16944, rs1143634, IL-Ira 86bp VNTR) and *HRAS1* (28bp-VNTR) gene

	**IL-1β (promoter region).**	**IL-1β (exon-5).**	**IL-1Ra.**	**HRAS**
**Type of polymorphism**	Single base C/T	Single base C/T	86-bp VNTR	28-bp VNTR
**Site of polymorphism**	–511	+3954	Intron-2	1kb downstream of HRAS gene
**PCR primers**				.
**Upstream :**	5'TGGCATTGATC TGGTTCATC-3'	5'GTTGTCATCAG ACTTTGACC-3'	5'CTCAGCAAC ACTCCTAT-3'	5'GCTCCTGGCCTC GGGAAGTCTAT3'
**Downstream:**	5'GTTTTAGGAAT CTTCCCACTT-3'	5'-TTCAGTTCAT ATGGACCAGA-3'	5'TCCTGGTCTG CAGGTAA-3'	5'AGAGCTAGCAG GGCATGCCGCT3'
**PCR conditions:**				
**Denaturation :**	95˚C, 1 min	95˚C, 1 min	95˚C, 1 min	94˚C, 1 min
**Annealing:**	55˚C, 1 min	59˚C, 1 min	55˚C, 1 min	67˚C, 1 min
**Extension:**	72˚C, 1 min	72˚C, 1 min	72˚C, 1 min	72˚C, 1 min
**No. of cycles:**	35	35	35	30
**Digestion:**	(Ava I)	(Taq I)	86-bp VNTR	28-bp VNTR
**Allele size, bp:**	*C: 190+114<br>*T: 304	*E1: 135+114<br>*E2: 249	1: 240bp, 2: 325bp,<br>3:410bp , 4: 500bp,	Common alleles<br>A1:840bp ,A2:1300bp<br>A3:1900bp ,<br>A4:2350bp

Table S2: Il-1β gene polymorphism and their minor allele frequencies, and HWE p-values in cases and control

Gene	SNP _ID	Cases (n =150)			Controls (n = 187)		
		MA[a]	MAF[b]	HWE[c]*p-value*	MA[a]	MAF[b]	HWE[c]*p-value*
IL-1 β	**rs16944**	T	41.4%	0.165	T	37.6%	0.604
	**rs1143634**	E2	28%	0.578	E2	35.8%	0.339

Table S3: Frequency of IL1B promoter gene polymorphisms in different populations.

Population	Cases			Control			p-value	Reference
	CC	CT	TT	CC	CT	TT		
**Germany (Caucasian)**	124 (46%)	114 (42.4)	31 (11.6%)	88 (38.7%)	111 (48.9%)	28 (12.4%)	0.44	(Hefler *et al.*, 2005)
**Indian**	for both 107 (53.5%)		93 (46.5%)	For both 119 (59.5%)		81 (40.5%)	0.226	(Pooja *et al.*, 2012)
**UK(White Caucasians)**	339 (48.2%)	294 (41.8%)	70 (10%)	232 (47.4%)	206 (42.1%)	51 (10.4%)	0.75	(Balasubramanian *et al.*, 2006)
**Japan (Asian)**	60 (42.6)	67 (47.5)	14 (9.9)	87 (33.3)	135 (51.7)	39 (14.9)	NS	(Smith, 2004)
**China (Asian)**	129 (20.4%)	305 (48.3%)	197 (31.3%)	101 (27.6%)	170 (46.6%)	94 (25.8)	0.19	(Liu *et al.*, 2006)
**Jordanian**	56 (37.1%)	65 (43.0%)	30 (19.9%)	74 (39.8%)	84 (45.2%)	28 (15%)	0.52	Our study

Table S4: Frequency of IL1B exon 5 gene polymorphisms in different populations.

**Population**	Cases			Control			p-value	Reference
	E1/E1	E1/E2	E2/E2	E1/E1	E1/E2	E2/E2		
**Germany (Caucasian)**	159 (59.1%)	97 (36%)	13 (4.9%)	119 (52.4%)	99 (43.6%)	9 (4%)	0.04	(Hefler *et al.*, 2005)
**Indian**	for both 53 (26.5%		147 (73.5%)	For both 24 (12%)		176 (88%)	0.226	(Pooja *et al.*, 2012)
**UK(White Caucasians)**	410 (59.3%)	242 (35%)	39 (5.6%)	231 (55%)	167 (39.8%)	22 (5.2%)	0.29	(Balasubramanian *et al.*, 2006)
**Tunisia African**	116 (38%)	132 (43.3%)	57 (18.6%)	83 (41.5%)	82 (41%)	35 (17.5%)	0.07	(Snoussi *et al.*, 2005)
**Jordanian**	78 (52.7%)	57 (38.5%)	13 (8.8%)	74 (39.6%)	92 (49.2%)	21 (11.2%)	0.057	Our study

Table S5: Genetic association studies of IL-1Ra gene polymorphisms in different populations.

**Population**	Cases	Control	Association based on *P-value*	Reference
**Indian**	n=100	n=200	N.S	(Konwar *et al.*, 2009)
**Korean**	n=586	n=522	S.A	(Lee *et al.*, 2006)
**Caucasian**	n=269	n=228	N.S	(Hefler *et al.*, 2005)
**Jordanian**	n=150	n=187	S.A	Our study

N.S: non-significant, S.A: significantly associated.

Table S6: Frequency of HRAS1 gene polymorphisms in different populations

**Cases**			Control			p-value	Reference
**C/C**	C/R	R/R	C/C	C/R	R/R		
**572(79.7%)**	137(19.1%)	8(1.1)	618(77.6%)	169(21.2%)	11(1.2%)	NS	(Tamimi *et al.*, 2003)
**Meta analysis**						0.001	(Zhang and Yu, 2011)
**Meta analysis**						0.84	(Zhang and Yu, 2011)
**At the allelic level only**						0.022	This study
